# The Use of Specific Serological Biomarkers to Detect CaniLeish Vaccination in Dogs

**DOI:** 10.3389/fvets.2019.00373

**Published:** 2019-10-24

**Authors:** Carla Lima, Nuno Santarém, Javier Nieto, Javier Moreno, Eugenia Carrillo, Daniella Castanheira Bartholomeu, Lilian Lacerda Bueno, Ricardo Fujiwara, Célia Amorim, Anabela Cordeiro-da-Silva

**Affiliations:** ^1^i3S – Instituto de Investigação e Inovação em Saúde, Universidade do Porto, Porto, Portugal; ^2^IBMC – Instituto de Biologia Molecular e Celular, Universidade do Porto, Porto, Portugal; ^3^Faculdade de Farmácia da Universidade do Porto, Departamento de Ciências Biológicas, Porto, Portugal; ^4^WHO Collaborating Centre for Leishmaniasis, National Centre for Microbiology, Instituto de Salud Carlos III, Majadahonda, Spain; ^5^Departamento de Parasitologia, Instituto de Ciências Biológicas, Universidade Federal de Minas Gerais (UFMG), Belo Horizonte, Brazil

**Keywords:** leishmaniosis, *Leishmania*, vaccination, diagnostic, serology

## Abstract

Canine leishmaniosis (CanL) prevention in the Mediterranean basin is considered essential to stop human zoonotic visceral leishmaniasis. In this context, vaccination of dogs is expected to have a significant impact in disease control. CaniLeish® (Virbac Animal Health) is one of a few CanL vaccines that are at this moment licensed in Europe. This vaccine contains purified excreted-secreted proteins of *Leishmania* having several antigens/immunogens with potential to influence serological response. Therefore, it is important to know if CaniLeish vaccination increased the diagnostic challenges associated with conventional serology, limiting the value of some antigens. To address this 20 dogs from a cohort of 35 healthy dogs that were vaccinated, maintained indoor for 1 month and then returned to their natural domiciles for 2 years. After this period, they were re-called to evaluate their clinical/parasitological condition and assess the evolution of seroreactivity against different antigens: soluble promastigote *Leishmania* antigens (SPLA), recombinant protein *Leishmania infantum* cytosolic peroxiredoxin, recombinant protein K39 (rK39), recombinant protein K28 and recombinant kinesin degenerated derived repeat using ELISA. Two years after vaccination all vaccinated non-infected animals were seropositive for SPLA. For the other antigens the serological profile was indistinguishable from non-infected animals. Moreover, vaccinated animals presented a characteristic relative serological profile, with higher normalized serological response to SPLA than rK39. This fact enabled to distinguish with sensitivity 92.3% and specificity 95.4%, vaccinated non-infected dogs from infected and non-infected dogs. Ultimately, relative serological profile enabled the detection of healthy vaccinated animals enabling more accurate serological surveys.

## Introduction

Canine leishmaniosis (CanL) is a vector-borne zoonotic disease, caused by protozoan parasites of the genus *Leishmania*, present in four continents impacting veterinary and public health (1–3). Moreover, the prevalence of human zoonotic visceral leishmaniasis (ZVL) and CanL, in endemic areas, are associated (4–6). Although early diagnosis, treatment, vector, and reservoir control are part of surveillance programs to control CanL (3, 7, 8), vaccination is considered an important tool to prevent the human and canine disease (9–11). Currently, in Europe, there are two available canine vaccines: CaniLeish® and LetiFend® (8, 12). The CaniLeish vaccine contains excreted/secreted antigens purified from the culture supernatant of *Leishmania infantum* promastigotes. The proteomic studies on excreted/secreted antigens confirm the existence of several proteins that are not exclusively secreted, being also present in parasite preparations (13, 14). Therefore, as CaniLeish induces Th1 cell-mediated response and production of IgG1 and IgG2 antibodies, seroreactivity due to natural infection or due to immunoprophylaxis is difficult to distinguish as cross-reactivity to several *Leishmania* antigens can occur (8, 15). Therefore, considering that CaniLeish is now available for over 7 years and vaccine-induced anti-*Leishmania* antibodies can be detectable for months after administration, it is important to evaluate if vaccine-induced seroconversion might represent a problem in surveillance and control programs (15). To address this possibility, serum of CaniLeish vaccinated dogs from a previous study (8), that involved the vaccination of 20 dogs from a cohort of 35 naive dogs and a subsequent 2 years follow up, was used to evaluate the evolution of seroreactivity to diagnosis relevant antigens like SPLA (soluble promastigote *Leishmania* antigens), rK39 and other molecules of high serological value like the kinesins constructs rK28 (16), recombinant kinesin degenerated derived repeat (rKDDR) (17) or recombinant protein *Leishmania infantum* cytosolic peroxiredoxin (LicTXNPx) (18) using enzyme-linked immunosorbent assay (ELISA). With the exception of LicTXNPx, a peroxiredoxin associated to the protection of the parasites from oxidative stress (19) all the recombinant proteins used are synthetic peptides containing or enriched in immunodominant epitopes. The rK39 is a repetitive immunodominant epitope from a kinesin-related protein conserved in viscerotropic *Leishmania* (20). The kDDR is a 39 amino acid repetitive sequence originated from an originated from an *in-silico* epitope prediction analysis (17). The rK28 is a synthetic construct created by fusing multiple tandem repeat sequences of *Leishmania donovani* from haspb1 and k39 kinesin genes to the ORF of haspb2 (21).

## Materials and Methods

### Animals

The study from which the serological samples originated involved cohort of 35 healthy dogs was studied for 2 years (8). Previously, parasitological (PCR from Bone Marrow and culture) and serological tests Indirect Fluorescence Antibody Test (IFAT) were performed to confirm the absence of *Leishmania* infection. Twenty dogs were vaccinated (V) with CaniLeish® (standard formulation) and 15 non-vaccinated animals were included as controls (NV). Primo-vaccination program was done in 3 doses with 21 days of interval each. After the last dose dogs were housed in kennels with insecticide nets for 1 more month. After this period the animals returned to their domiciles. A booster vaccination was performed 1 year after the third dose of primo-vaccination.

### Sampling

Samples were collected at two-time points as described in the original study (8): M1 (1 month after the administration of the last dose of primo-vaccination, at the end of their period in the kennel) and M25 (25 months after the last dose of primo-vaccination). IFAT (Indirect Fluorescence Antibody Test), PCR and culture assays were performed in the two-time points (8). Clinical surveillance was also done to evaluate clinical manifestations compatible with CanL. At the end of the study, the cohort was sub-divided in 4 groups concerning PCR and clinical evaluation: C- (non-vaccinated non-infected animals), C+ (non-vaccinated infected animals), V- (vaccinated non-infected animals), and V+ (vaccinated infected animals).

### Antigens

For SPLA, *Leishmania* promastigotes were cultivated as previously described (22). Parasites with 5 days of culture were then washed 3 times with phosphate-buffered saline (PBS) pH 7.4 and centrifuged at 3,500 × g, 10 min, at 4°C. The pellet was then suspended at a concentration of 10^8^ parasites/ml in PBS containing 1 mM phenylmethylsulfonyl fluoride protease inhibitor and submitted to 10 freeze-thaw cycles for rupture of the parasites. This suspension was centrifuged at 13,000 × g, 30 min, at 4°C and the supernatant was recovered, quantified by DC (detergent compatible)^TM^ Protein Assay (BioRad, Germany), and stored at −80°C in single-use aliquots. The recombinant protein *Lic*TXNPx was purified by affinity chromatography on a Ni-NTA column (Qiagen) as described in previous reports (23) and obtained as a recombinant protein containing six histidine residues at its N-terminal. The r*Lic*TXNPx was quantified and stored at −80°C in single aliquots. The rK39 and rK28 lyophilized antigens, obtained from Dr. Steven Reed, from Infectious Disease Research Institute (Seattle, USA) were suspended in deionized and 0.22 μm membrane-filtered H_2_O, quantified and stored at −80°C in single use-aliquots. The recombinant protein rKDDR, provided by Dr. Ricardo Fujiwara, from Universidade Federal de Minas Gerais (Belo Horizonte, Brasil), was quantified and stored at −80°C in single aliquots.

### Evaluation of Seroreactivity

The serological reactivity in the canine samples was evaluated by ELISA using each of the 5 different antigens: SPLA, LicTXNPx, rK39, K28, and rKDDR. Ninety-six-well flat-bottomed microtiter plates (Greiner Bio-One) were coated with 50 μl of 0.1 M carbonate buffer, pH = 9.6, with 10 μg/ml of SPLA, 3 μg/ml of LicTXNPx, 1 μg/ml of rK39, 4 μg/ml of rK28, and 3 μg/ml of rKDDR. Plates were incubated overnight at 4°C and blocked with 200 μl of PBS-low-fat-milk 3% at 37°C for 1 h. Next, plates were washed with PBS-Tween 0.05% (PBS-T), and the sera, positive and negative controls diluted 1:1,500 in PBS-T-low-fat-milk 1%, were dispensed in triplicate (100 μl/well) and incubated at 37°C for 30 min. Wells that have the antigen, that are blocked and only incubated with the secondary antibody are the blank situation that works as a negative control. As an internal positive control we used a mixture of sera from several highly seropositive positive CanL dogs. This pool was made aliquoted and used for the duration of the study. When a batch is finishing a new blend of pooled CanL sera is done and then compared to the old batch to confirm the new internal positive control. Consequently, the OD obtained for each antigen is characteristic of the positive control and in conjunction with the negative control above mentioned enables the required quality control for each assay, assuring greater reproducibility. After a washing step, 100 μl/well of secondary antibody—anti-dog IgG conjugated to horseradish peroxidase (Sigma)—diluted 1:1176.5, was added and the plates were incubated at 37°C for 30 min. Plates were washed and incubated with 0.5 mg/ml of o-phenylenediamine dihydrochloride (Sigma) for 10 min in the dark. The reaction was stopped with 50 μl/well of HCl 3 M. Absorbance was read at 492 nm in an automatic reader (Synergy 2, BioTek Instruments, Inc., Vermont). All samples and antigens were assayed in triplicate in at least two independent assays.

### Statistical Analysis

Receiver operating characteristic (ROC) curves were generated using sera from two distinct groups of animals: 29 dogs with confirmed CanL living in geographical regions of Portugal where CanL is endemic and 121 non-infected dogs originated from a non-endemic region from Portugal. A 95% confidence interval (95% CI) for the area under the ROC curve was considered. Cut-off values were inferred through these curves for each antigen (by choosing the best compromise between sensitivity and specificity associated with the ROC curve). The optical densities (OD) of each sample were normalized by division with the corresponding antigen cut-off. These normalized values were used to assess the ratio between the antigens. The logarithm to base 10 of normalized optical densities was used for comparative graphical representation. The values of sensitivity (Se), specificity (Sp), positive predictive value (PPV), and negative predictive value (NPV) were calculated for each antigen ratio. The ROC curves, Unpaired *t* test with Welch's correction, One-way ANOVA, Mann-Whitney, and Kruskal-Wallis tests were performed using GraphPad Prism 5 software (GraphPad Software, USA).

## Results

### Evaluation of Seroreactivity at M1 Time Point

**Figure 1:**
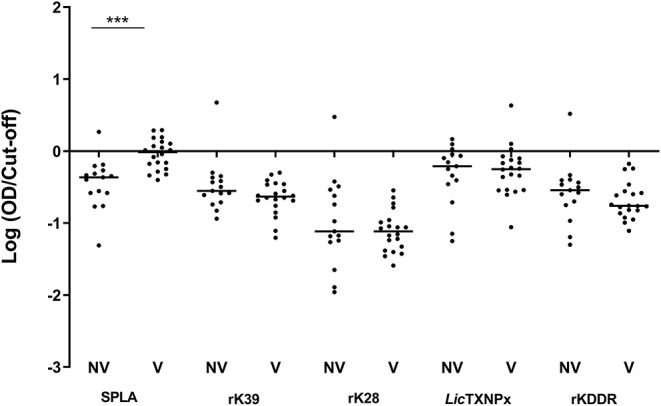
Seroreactivity at M1 for non-vaccinated (NV) and vaccinated (V) dogs to the different antigens. Graphical representation of the median serological response to SPLA, rK39, rK28, LicTXNPx, and rKDDR. Results are expressed as the logarithm of the optical density values normalized by the cut-off for each antigen. Each dot represents the average of at least two independent experiments performed in triplicate. Statistical analysis was done by Unpaired *t* test with Welch's correction. One level of significance is represented: “extremely significant” (^***^0.0001 ≤ *P* < 0.001).

ROC curves were determined for all antigens and cut-off values inferred (Supplementary Table 1). One month after primo-vaccination, the reactivity to SPLA was significantly increased (*P* = 0.0007) in vaccinated dogs (V) when comparing to non-vaccinated (NV) (Figure 1). In fact, from the 20 vaccinated animals, 10 presented serology above the defined cut-off for SPLA at M1 time point. The reactivity to rK39, rK28, *Lic*TXNPx, and rKDDR antigens for V and NV groups in this time point was not significantly different (*P* < 0.05).

### Evaluation of Seroreactivity at M25 Time Point

**Figure 2:**
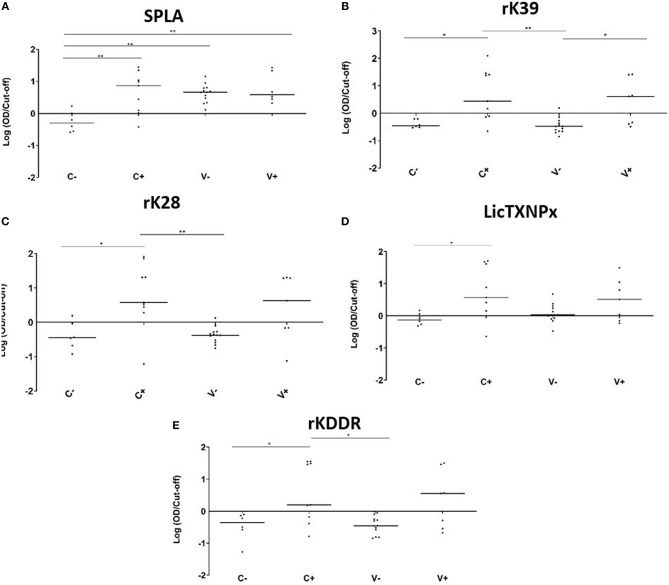
Seroreactivities at M25 in C-, C+, V-, and V+ groups against SPLA, rK39, rK28, LicTXNPX, and rKDDR antigens. Graphical representation of the median serological response to SPLA **(A)**, rK39 **(B)**, rK28 **(C)**, LicTXNPx **(D)**, and rKDDR **(E)**. Results are expressed as the logarithm of the optical density values normalized by the cut-off for each antigen. Each dot represents represent the average of at least two independent experiments performed in triplicate. Statistical analysis was done by one-way ANOVA and Kruskal-Wallis tests. Three levels of statistical significance are represented: “significant” (^*^0.01 ≤ *P* < 0.05), and “very significant” (^**^0.001 ≤ *P* < 0.01).

The V-, V+, C-, and C+ reactivity to the 5 antigens tested, was analyzed at the M25 time point (Figure 2). In non-vaccinated dogs, all tested antigens were significantly more recognized by the infected animals when compared to the non-infected group (Figure 2). When considering just vaccinated animals, only rK39 was able to significantly discriminate infected from non-infected animals (*P* = 0.0225). The antigen recognition in V- and C- was not significantly different for the recombinant antigens. The same was not true for SPLA that was significantly more recognized in V- (*P* = 0.0015) (Figure 2A). In fact, 25 months after immunization all vaccinated animals were seropositive to SPLA. Nine V- animals were also seropositive to LicTXNPx being the second antigen with most seropositive animals in this group (Figure 2D). For the other antigens tested, the V- group contained at most one seropositive animal. Concerning the infected animals, the serological response to the different antigens in the V+ and C+ groups was not significantly different for all the antigens tested. Still, with the exception of SPLA, the percentage of seropositive animals was higher in C+ when compared to V+.

### Seropositivity Ratios for Detection of Vaccinated Non-infected Dogs

**Figure 3:**
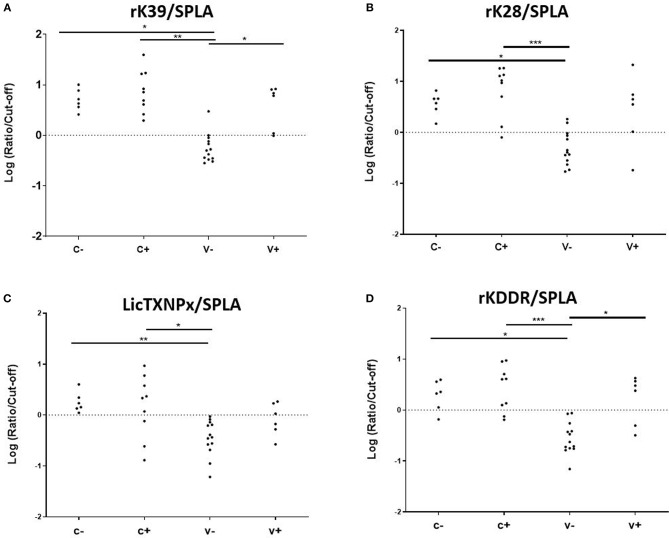
Seroreactivities at M25 in C-, C+, V-, and V+ groups for the antigen/SPLA ratios. Graphical representation of the response to rK39/SPLA **(A)**, rK28/SPLA **(B)**, LicTXNPx/SPLA **(C)**, and rKDDR/SPLA **(D)**. Results are expressed as the logarithm of the optical density values normalized by the cut-off for each antigen. Each dot represents represent the average of at least two independent experiments performed in triplicate. Statistical analysis was done by one-way ANOVA and Kruskal-Wallis tests. Three levels of statistical significance are represented: “significant” (^*^0.01 ≤ *P* < 0.05), “very significant” (^**^0.001 ≤ *P* < 0.01), and “extremely significant” (^***^0.0001 ≤ *P* < 0.001).

To understand if the pattern of seropositivity to SPLA and seronegativity to the recombinant antigens had some predictive characteristic value (Table 1), the ratios between the normalized responses to each antigen (rK39, rK28, *Lic*TXNPx, and rKDDR) and SPLA were calculated for V-, V+, C- and C+ groups (rK39/SPLA, rK28/SPLA, *Lic*TXNPx/SPLA, rKDDR/SPLA) and ROC curves were calculated (Supplementary Table 2). A cut-off value for each ratio was inferred by the respective ROC curve and used to normalize the data, which was then logarithmized (Figure 3). As observed by the ROC curves and by the graphical representation of the ratios rK39/SPLA, rK28/SPLA, *Lic*TXNPx/SPLA, and rKDDR/SPLA, the ratio that performed better was rK39/SPLA. The ratio rK39/SPLA represented the best compromise between Se (92.3%) and Sp (95.4%), and consequently, the best compromise between positive predictive value (92.8%) and negative predictive value (95.6%) (Supplementary Table 3).

**Table 1:** Comparison of seropositivity observed for SPLA with seronegativity observed for the antigens rK39, rK28, LicTXNPx, rKDDR, LAM, and RPM at M25 time point.

	**SPLA/rK39**	**SPLA/rK28**	**SPLA/LicTXNPx**	**SPLA/rKDDR**
V-	92.3_(12/13)_	92.3_(12/13)_	38.5_(5/13)_	100_(13/13)_
V+	42.8_(3/7)_	42.8_(3/7)_	28.6_(2/7)_	42.8_(3/7)_
C-	33.3_(2/6)_	16.7_(1/6)_	0.0_(0/6)_	33.3_(2/6)_
C+	22.2_(2/9)_	0.0_(0/9)_	11.1_(1/9)_	22.2_(2/9)_
Total^*^	31.8_(7/22)_	18.2_(4/22)_	13.6_(3/22)_	31.8_(7/22)_

* *All non V- dogs*.

## Discussion

In the reported study, seropositivity to 5 different antigens was evaluated in a cohort of vaccinated/non-vaccinated animals with CaniLeish that were followed up at 1 and 25 months after Primo-vaccination. At a serological level, after 1 month of vaccination, the group of vaccinated animals (V) was significantly more reactive to SPLA suggesting an early seroconversion that was maintained. In fact, the response of the V- to SPLA was indistinguishable from the V+ and C+. This reactivity to SPLA resulting from vaccination might translate into cross-reactivity to other tests like IFAT or DAT that rely on the identification of total parasite antigens. In fact, at M25 time point, all vaccinated animals tested positive to IFAT (Supplementary Table 3). This observation was also reported by Moreno et al. (24) and Oliva et al. (8). This is not surprising as the secreted material from the parasite contains several surface and intracellular antigens that can be recognized during a normal infection (14, 25). On the contrary, the recombinant antigens tested were not recognized significantly by the non-infected vaccinated group (Figure 2). The antigen *Lic*TXPx was an exception, presenting a distinct behavior with eight seropositive from 13 infected dogs. A possible explanation for this unique behavior might be the possibility of undetected asymptomatic infections. This antigen is a potential serological marker in detecting asymptomatic infection and in early seroconversion (18, 26). Therefore, we cannot rule out that the increase in reactivity to this antigen in V- group might be due to a low intensity (undetected by PCR) asymptomatic infection. Considering the serology results obtained it is clear that CaniLeish vaccination abrogates the usefulness of SPLA and other techniques that rely on the detection of parasites like, IFAT, DAT. Recombinant antigens were not able to detect vaccinated healthy animals not enabling the detection of vaccinated animals in field surveys.

Considering that the vaccine-associated reactivity to SPLA was the only measure of exposure to the vaccine, seropositivity to SPLA, and seronegativity to any given antigen would be a serological profile characteristic of vaccinated non-infected animals as is depicted in Table 1. In V- group, with the exception for *Lic*TXNPx, the pattern of SPLA seropositive and seronegative to recombinant proteins —rK39, rK28, rKDDR, *Lic*TXNPx,– was observed in at least 11 of the 13 animals (84.6%), and in all 13 dogs for SPLA+/rKDDR- (100%). Nonetheless, this profile although observed in the V- group was also present in a lesser extent (13.6–31.8%, dependent on the antigen), in the C- group. Considering this scenario, a different approach was tested. We had already reported that the relationship between the seropositivity to rK39 and SPLA presented a pattern that could be predictive of disease associated seropositivity (27), this was once again confirmed in the context of vaccination (Figure 3). In fact, by performing the ratios between the four antigens and SPLA (rK39/SPLA, rK28/SPLA, *Lic*TXNPx/SPLA, rKDDR/SPLA) and respective ROC curves, we could for the first time distinguish the V- (vaccinated healthy animals) from the other groups studied (V+, C-, and C+). In fact, the rK39/SPLA ratio (with 92.3% of Se and 95.4% of Sp) performed the best in distinguish V- animals, which is more informative than the cumulative seropositivity to the individual antigens.

In conclusion, we reported the serological response to different antigens in the context of CaniLeish vaccination confirming that SPLA is recognized by vaccinated animals limiting the use of IFAT, DAT and total antigen ELISA. We also confirm that the vaccine does not induce significant serological responses to rK39, rK28, LicTXNPx, and rKDDR. We also presented a new approach that enabled the identification of seropositive vaccinated healthy animals from vaccinated parasitized animals, non-vaccinated parasitized animals and non-vaccinated healthy animals, using the relation between the seroreactivities of two different antigens (SPLA and rK39) as a form of DIVA (Differentiating Infected from Vaccinated Animals) for CaniLeish. Ultimately, the evaluation of specific serological profiles associated with quantitative serology might enable the discrimination of not only vaccinated animals, as was proposed, but also contribute to finding specific serologic imprints associated do symptomatic and asymptomatic disease.

## Data Availability Statement

All datasets generated for this study are included in the manuscript/Supplementary Files.

## Ethics Statement

All study procedures were approved by the National Authorities in Catalonia (Spain). The study design and technical protocol of investigations were approved by the Department of Biodiversity and the Environment of the Government of Catalonia under number 6760 in accordance with Spanish law on the protection of animals used for experimentation and other scientific purposes (Royal Decree 1201/2005 and Law 32/2007). The Spanish legislation is a transposition of Directive 86/609/EEC.

## Author Contributions

NS and CL performed the ELISAS. NS, CL, CA, and AC designed the study. NS, CL, and AC analyzed the data and wrote the paper. JN, JM, and EC were involved in the animal vaccination and characterization of the animals. DB, LB, and RF were involved in the production and disponibilization of rKDDR. All authors read and approved the final manuscript.

### Conflict of Interest

The authors declare that the research was conducted in the absence of any commercial or financial relationships that could be construed as a potential conflict of interest.
