# Supplementary material for: The Use of Specific Serological Biomarkers to Detect CaniLeish Vaccination in Dogs
Source: Front Vet Sci. 2019 Oct 24;6:373. doi: 10.3389/fvets.2019.00373 (PMC6821643; doi:10.3389/fvets.2019.00373)
Supplement: Supplementary file 1 [file Table_1.DOCX]

Supplementary Material

# Supplementary Table 1 – Characterization and distribution of the cohorts in the vaccination study.

|  | **Month** | **IFAT titer** | **IFAT result** | **PCR Bone marrow** | **Culture Bone marrow** | **VL disease at M24** |
| --- | --- | --- | --- | --- | --- | --- |
| **CONTROL** | M1 |  1/40 | NEG | ND | ND | VL |
| **CONTROL** | M24 | 1/80 | POS | - | contaminated | VL |
| **CONTROL** | M1 |  1/40 | NEG | ND | ND | VL |
| **CONTROL** | M24 | 1/80 | POS | + | - | VL |
| **CONTROL** | M1 |  1/40 | NEG | ND | ND | VL |
| **CONTROL** | M24 | 1/80 | POS | - | - | VL |
| **CONTROL** | M1 |  1/40 | NEG | ND | ND | VL |
| **CONTROL** | M24 | 1/640 | POS | + | + | VL |
| **CONTROL** | M1 |  1/40 | NEG | ND | ND | VL |
| **CONTROL** | M24 | 1/80 | POS | + | + | VL |
| **CONTROL** | M1 |  1/40 | NEG | ND | ND | VL |
| **CONTROL** | M24 | 1/80 | POS | + | + | VL |
| **CONTROL** | M1 |  1/40 | **NEG** | ND | ND | VL |
| **CONTROL** | M24 | 1/160 | **NEG** | - | + | VL |
| **CONTROL** | M1 |  1/40 | NEG | ND | ND | - |
| **CONTROL** | M18 | 1/5120 | POS | + | + | - |
| **CONTROL** | M1 |  1/40 | NEG | ND | ND | - |
| **CONTROL** | M18 | 1/5120 | POS | + | + | - |
| **CONTROL** | M1 |  1/40 | NEG | ND | ND | - |
| **CONTROL** | M24 |  1/40 | NEG | - | - | - |
| **CONTROL** | M1 |  1/40 | NEG | ND | ND | - |
| **CONTROL** | M24 |  1/40 | NEG | - | - | - |
| **CONTROL** | M1 |  1/40 | NEG | ND | ND | - |
| **CONTROL** | M24 |  1/40 | NEG | - | - | - |
| **CONTROL** | M1 |  1/40 | NEG | ND | ND | - |
| **CONTROL** | M24 |  1/40 | NEG | - | - | - |
| **CONTROL** | M1 |  1/40 | NEG | ND | ND | - |
| **CONTROL** | M24 |  1/40 | NEG | - | - | - |
| **CONTROL** | M1 |  1/40 | NEG | ND | ND | - |
| **CONTROL** | M24 |  1/40 | NEG | - | - | - |
| **VACCINATED** | M1 | 1/40 | DOUBT | ND | ND | VL |
| **VACCINATED** | M24 | 1/320 | POS | + | - | VL |
| **VACCINATED** | M1 | 1/40 | DOUBT | ND | ND | VL |
| **VACCINATED** | M24 | 1/2560 | POS | + | + | VL |
| **VACCINATED** | M1 |  1/40 | NEG | ND | ND | VL |
| **VACCINATED** | M24 | 1/320 | POS | + | + | VL |
| **VACCINATED** | M1 | 1/80 | POS | ND | ND | - |
| **VACCINATED** | M24 | 1/160 | POS | + | - | - |
| **VACCINATED** | M1 | 1/80 | POS | ND | ND | - |
| **VACCINATED** | M24 | 1/160 | POS | + | - | - |
| **VACCINATED** | M1 |  1/40 | NEG | ND | ND | - |
| **VACCINATED** | M24 | 1/2560 | POS | + | + | - |
| **VACCINATED** | M1 | 1/40 | DOUBT | ND | ND | - |
| **VACCINATED** | M24 | 1/640 | POS | + | - | - |
| **VACCINATED** | M1 | 1/80 | POS | ND | ND | - |
| **VACCINATED** | M24 | 1/320 | POS | - | - | - |
| **VACCINATED** | M1 | 1/160 | POS | ND | ND | - |
| **VACCINATED** | M24 | 1/320 | POS | - | - | - |
| **VACCINATED** | M1 | 1/40 | DOUBT | ND | ND | - |
| **VACCINATED** | M24 | 1/160 | POS | - | - | - |
| **VACCINATED** | M1 | 1/80 | POS | ND | ND | - |
| **VACCINATED** | M24 | 1/320 | POS | - | - | - |
| **VACCINATED** | M1 | 1/80 | POS | ND | ND | - |
| **VACCINATED** | M24 | 1/160 | POS | - | - | - |
| **VACCINATED** | M1 | 1/80 | POS | ND | ND | - |
| **VACCINATED** | M24 | 1/160 | POS | - | - | - |
| **VACCINATED** | M1 | 1/80 | POS | ND | ND | - |
| **VACCINATED** | M24 | 1/80 | POS | - | - | - |
| **VACCINATED** | M1 | 1/40 | DOUBT | ND | ND | - |
| **VACCINATED** | M24 | 1/160 | POS | - | - | - |
| **VACCINATED** | M1 |  1/40 | NEG | ND | ND | - |
| **VACCINATED** | M24 | 1/160 | POS | - | - | - |
| **VACCINATED** | M1 | 1/80 | POS | ND | ND | - |
| **VACCINATED** | M24 | 1/160 | POS | - | - | - |
| **VACCINATED** | M1 | 1/40 | DOUBT | ND | ND | - |
| **VACCINATED** | M24 | 1/320 | POS | - | - | - |
| **VACCINATED** | M1 |  1/40 | NEG | ND | ND | - |
| **VACCINATED** | M24 | 1/40 | DOUBT | - | - | - |
| **VACCINATED** | M1 |  1/40 | NEG | ND | ND | - |
| **VACCINATED** | M24 | 1/80 | POS | - | - | - |
