# Supplementary material for: The Use of Specific Serological Biomarkers to Detect CaniLeish Vaccination in Dogs
Source: Front Vet Sci. 2019 Oct 24;6:373. doi: 10.3389/fvets.2019.00373 (PMC6821643; doi:10.3389/fvets.2019.00373)
Supplement: Supplementary file 2 [file Table_2.DOCX]

Supplementary Material

# Supplementary Table 2 ROC curve analysis for the 5 antigens used.

|  | **SPLA** | **rK39** | ***Lic*TXNPx** | **rK28** | **rKDDR** |
| --- | --- | --- | --- | --- | --- |
| **AUC** | 1.000 | 1.000 | 0.84 | 1.000 | 1.000 |
| **Cut-off** | 0.075 | 0.127 | 0.040 | 0.175 | 0.098 |
| **Se %** | 100 | 100 | 93.1 | 100 | 100 |
| **Sp %** | 100 | 100 | 94.2 | 100 | 100 |
| **FP %** | 0 | 0 | 5.8 | 0 | 0 |
| **FN %** | 0 | 0 | 6.9 | 0 | 0 |
| **PPV %** | 100 | 100 | 80.6 | 100 | 100 |
| **NPV %** | 100 | 100 | 98.4 | 100 | 100 |

AUC: area under the curve; Se: sensitivity; Sp: specificity; FP: false positives; FN: false negatives;

PPV: positive predictive value; NPV: negative predictive value.
