# Supplementary material for: The Use of Specific Serological Biomarkers to Detect CaniLeish Vaccination in Dogs
Source: Front Vet Sci. 2019 Oct 24;6:373. doi: 10.3389/fvets.2019.00373 (PMC6821643; doi:10.3389/fvets.2019.00373)
Supplement: Supplementary file 3 [file Table_3.DOCX]

Supplementary Material

|  | rK39/SPLA | rK28/SPLA | *Lic*TXNPx/SPLA | rKDDR/SPLA |
| --- | --- | --- | --- | --- |
| **Se (%)** | 92.3 | 84.6 | 100 | 100 |
| **Sp (%)** | 95.4 | 90.9 | 68.2 | 72.7 |
| **PPV (%)** | 92.8 | 86.7 | 100 | 100 |
| **NPV (%)** | 95.6 | 91.7 | 81.5 | 81.5 |
| **Cut-off** | 0.144 | 0.200 | 0.793 | 0.316 |
| **AUC** | 0.975 | 0.916 | 0.825 | 0.927 |

**Supplementary Table 3** - ROC curve analysis for the six different antigen ratios: rK39/SPLA, rK28/SPLA, *Lic*TXNPx/SPLA, rKDDR/SPLA,
